# Editorial: Modulation of microglia reactivity and plasticity in CNS injury

**DOI:** 10.3389/fncel.2023.1277382

**Published:** 2023-09-12

**Authors:** Zhenyan He, Hailiang Tang, Jiping Tang, Xuying He, Ningbo Xu, Devin W. McBride

**Affiliations:** ^1^Department of Neurosurgery, Affiliated Tumor Hospital of Zhengzhou University, Zhengzhou, China; ^2^Department of Neurosurgery, Huashan Hospital, Fudan University, Shanghai, China; ^3^Department of Physiology and Pharmacology, Basic Sciences, School of Medicine, Loma Linda University, Loma Linda, CA, United States; ^4^Department of Interventional Radiology, Zhujiang Hospital, Southern Medical University, Guangzhou, China; ^5^The Vivian L. Smith Department of Neurosurgery, The University of Texas Health Science Center at Houston, Houston, TX, United States

**Keywords:** immune cell reactivity, microglia polarization, stroke, traumatic brain injury, hematoma resolution

Injury to the central nervous system (CNS) includes a variety of traumatic events, such as spinal cord injury and traumatic brain injury, but also brain diseases, such as stroke, brain aneurysms and arteriovenous malformations. CNS injuries are a leading cause of death and disability worldwide. Regardless of the type of CNS injury, microglia are at the fore-front of the CNS immune response to protect and repair brain damage.

Microglia, the resident macrophages in the brain, are considered among the most versatile cells in the brain; microglia are a key regulator in CNS homeostasis. During disease, microglia can become pro-inflammatory or anti-inflammatory, depending on its reactivity and polarization. In fact, Microglia have a variety of phenotypes they can become (from classically activated (M1) phenotype to alternatively activated phenotypes (M2a, M2b, M2c, M4), a process termed polarization) which allows them to functionally adapt to their ever-changing surroundings and be an important regulator within the brain. However, recently, the high reactivity or polarization of immune cells have shown to be connected to neurodevelopmental disorders, neuropsychiatric conditions, chronic pain, infectious diseases, and responses after sterile brain injury, particularly in stroke and traumatic brain injury.

In this Research Topic, the goal was to provide a collection of papers which the primary focus of gaining a better and deeper understanding of the reactivity and polarization of microglia and the roles in regulating CNS injury.

The review by Zhang et al. describes in detail the various intercellular communication mechanisms which rely on microglia. The review article focuses on stroke, but the information is applicable to various CNS injuries. Of note, the authors introduce the different microglia phenotypes and provide evidence from literature when microglia switching occurs. In detail, the authors describe the interactions microglia have with neurons and astrocytes, including the differences in the interactions with these two cells for M1 (pro-inflammatory) and M2 (anti-inflammatory) microglia. Microglia are also key cells for the interactions with other brain cells, but also aid in gate-keeping for circulating immune cells.

The article by Yu et al. explored the role of metformin in microglia polarization after intracerebral hemorrhage. Using a mouse model of intracerebral hemorrhage, the authors observed that metformin treatment inhibits the pro-inflammatory polarization of microglia, thereby alleviating neurological dysfunction. Moreover, metformin altered the microbiota composition which corresponded with improved intestinal barrier function. Interestingly, fecal transplantation from metformin-treated mice provided a similar beneficial effect against intracerebral hemorrhage as oral metformin-treatment, suggesting that metformin's treatment effects may rely on the gut microbiota's interactions with metformin.

Wei et al. tested if dexmedetomidine could alleviate pyroptosis after subarachnoid hemorrhage. Using rodents, the authors observed that dexmedetomidine prevented microglial pyroptosis during early brain injury after subarachnoid hemorrhage, leading to less pro-inflammation and more anti-inflammation which corresponded with improve neurological performance. However, whether or not microglia polarization occurred, or if there was only less microglia reactivity, could be the focus of future studies.

The role of microglia activation following spinal cord injury was examined by Hu et al.. Recovery following spinal cord injury can be attenuated by damage to the primary motor cortex neurons. The authors report that microglia within the primary motor cortex are activated after spinal cord injury. These activated microglia led to NLRP3-induced inflammation and neuronal damage. Treatment with minocycline was capable of inhibiting microglia activation in the motor cortex, leading to improved functional outcomes and less inflammation. Whether microglia in the primary motor cortex are only less reactive, or if polarization into anti-inflammatory phenotypes occurred, was not reported. As minocycline was administered orally, future studies would need to study if the microglia within the primary motor cortex are a key treatment target.

In the future, understanding the precise roles of microglia reactivity and polarization in neuroinflammation may yield therapeutic targets to mitigate the detrimental effects proinflammation and promote anti-inflammatory repair after CNS injury. However, microglia function should focus on the varieties of phenotypes rather than placing them into dichotomized types (i.e., resting vs. active, M1 vs. M2) (Paolicelli et al., 2022).

## Author contributions

ZH: Writing—review and editing. HT: Writing—review and editing. JT: Writing—review and editing. XH: Writing—review and editing. NX: Writing—review and editing. DM: Writing—review and editing, Writing—original draft.
